# Decoding the Chemical Signatures and Sensory Profiles of Enshi Yulu: Insights from Diverse Tea Cultivars

**DOI:** 10.3390/plants12213707

**Published:** 2023-10-27

**Authors:** Yating Guo, Yili Shen, Boya Hu, Huichun Ye, Haowei Guo, Qiang Chu, Ping Chen

**Affiliations:** Tea Research Institute, Zhejiang University, Hangzhou 310058, China; ytguo@zju.edu.cn (Y.G.); shenyili@zju.edu.cn (Y.S.); 3200101558@zju.edu.cn (B.H.); 3200101562@zju.edu.cn (H.Y.); hwguo@zju.edu.cn (H.G.); 0619363@zju.edu.cn (Q.C.)

**Keywords:** Enshi Yulu, tea cultivars, sensory traits, chemical profiles, flavor contributors

## Abstract

Enshi Yulu, a renowned Chinese steamed green tea, is highly valued for its unique sensory attributes. To enhance our comprehensive understanding of the metabolic variation induced by steaming fixation, we investigated the overall chemical profiles and organoleptic quality of Enshi Yulu from different tea cultivars (Longjing 43, Xiapu Chunbolv, and Zhongcha 108). The relationships between sensory traits and non-volatiles/volatiles were evaluated. A total of 58 volatiles and 18 non-volatiles were identified as characteristic compounds for discriminating among the three tea cultivars, and the majority were correlated with sensory attributes. The “mellow” taste was associated with L-aspartic acid, L-asparagine, L-tyrosine, L-valine, EGC, EC, and ECG, while gallic acid and theobromine contributed to the “astringent” taste. “Kokumi” contributors were identified as L-methionine, L-lysine, and GCG. Enshi Yulu displayed a “pure” and “clean and refreshing” aroma associated with similar volatiles like benzyl alcohol, δ-cadinene, and muurolol. The composition of volatile compounds related to the “chestnut” flavor was complex, including aromatic heterocycles, acids, ketones, terpenes, and terpene derivatives. The key contributors to the “fresh” flavor were identified as linalool oxides. This study provides valuable insights into the sensory-related chemical profiles of Enshi Yulu, offering essential information for flavor and quality identification of Enshi Yulu.

## 1. Introduction

Green tea, derived primarily from the tender leaves of tea plants (*Camellia sinensis*), is a popular unfermented beverage consumed worldwide. It is renowned for its delightful flavor, along with many health-promoting properties of antioxidants and nutrients such as protein, amino acids, and flavonoids. The decisive manufacturing process for the formation of green tea quality is the fixation that passivates enzymes [1], according to which green tea can be classified into two main categories: roasted and steamed green tea [2]. Enshi Yulu stands out as the only needle-like green tea that adopts steaming fixation preserved in Chinese history and is also one of the best-selling green tea categories globally, particularly in Asia [3]. However, despite its popularity, there is a lack of systematic research and comprehensive evaluation of the organoleptic quality and related chemical profiles of Enshi Yulu.

Previous research has highlighted the crucial role of the production process in determining tea quality, as it influences the composition of various metabolites [4,5,6]. For instance, drying green tea at 90 °C tends to produce a clean aroma while drying at 160 °C enhances a high-fire aroma. Drying temperatures between 90 °C and 160 °C can elicit a chestnut-like or bean-like aroma [5]. Unlike the common roasting fixation, the steaming fixation method allows for the penetration of vapors, facilitating rapid and uniform fixation, which shapes the composition of the substances that make up steamed green tea differently from the roasted ones [2]. Above 80% of the non-volatile compounds change significantly after the steaming fixation based on widely targeted metabolomic analysis [2]. Additionally, steaming leads to lower levels of EC, EGC, EGCG, and ECG compared to roasting [7], which are key flavor determinants contributing to bitterness and astringency taste in green tea [8]. Moreover, the volatile compositions of steamed green teas significantly differ compared to other green teas, with a higher proportion of volatile alcohols [4,7]. A study conducted on Japanese green teas has identified indole as a key odorant that imparts floral aroma to steamed green teas, while high levels of phenolic compounds, as well as Maillard-reaction products such as pyrazines and pyrrole, evoke toasty and woody notes in roasted tea [9]. The unique steaming fixation endows Enshi Yulu with pure and natural characteristics [6], including a soothing aroma and mellow taste. However, current studies on Chinese steamed green teas primarily focus on the variation among different green teas [4,7], manufacturing processes [2], or isolated sensory analysis [10]. There exists a noticeable gap in comprehensive and systematic research on chemical profiles responsible for the distinctive sensory quality of steamed green teas.

In addition, Chinese tea cultivars Longjing 43, Xiapu Chunbolv, and Zhongcha 108, with the characteristics of early budding and high tenderness, are the primary varieties in the current Enshi Yulu market [10,11,12]. Green teas made from fresh leaves of Zhongcha 108 and Longjing 43 have high freshness and soft flavor due to lower phenolic to amino acid ratios [11,13,14]. Notably, Longjing 43 and Zhongcha 108 boast elevated flavonoid glycoside levels alongside heightened amino acid content, culminating in green teas characterized by heightened freshness and reduced astringency [14]. However, most of these studies across various tea cultivars are based on roasted green tea. Therefore, the perception and exploration of the relationship between chemical profiles and flavor attributes of Enshi Yulu from different tea cultivars would pave the fundament for the enhancements and regulation strategies of steamed green tea quality.

In this study, Enshi Yulu samples produced from the three typical tea plant cultivars (Longjing 43, Xiapu Chunbolv, and Zhongcha 108) were utilized to investigate the overall chemical profiles associated with organoleptic quality. The disparities of volatile and non-volatile components, as well as the sensory attributes among three tea cultivars, were determined. Additionally, the chemical factors contributing to the characteristic taste and aroma in the three Enshi Yulu teas were identified. This study marks the first comprehensive exploration of the flavor and chemical contributors specific to Enshi Yulu and enhances the understanding of the metabolic variation induced by steaming fixation and various cultivars.

## 2. Results

### 2.1. Sensory Traits of Enshi Yulu from Three Cultivars

**Figure 1:**
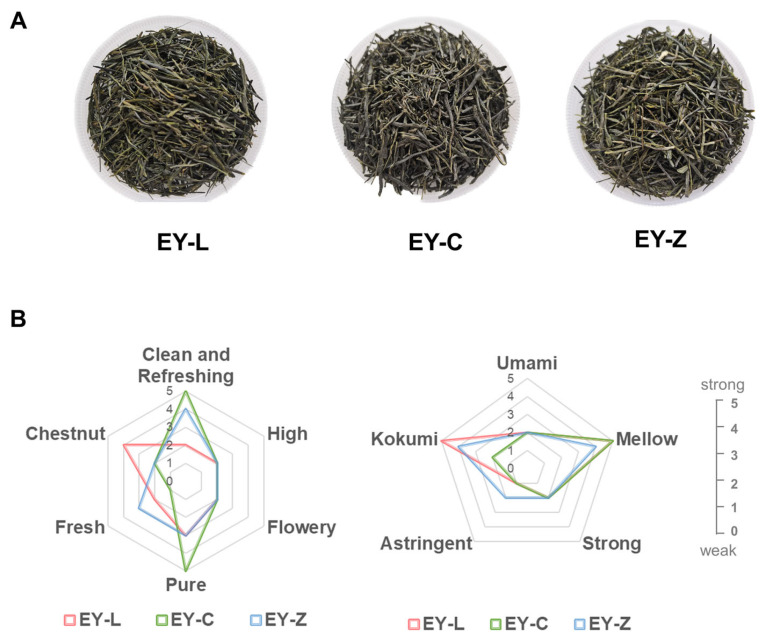
Enshi Yulu tea samples and sensory evaluation. (**A**) Appearance of three Enshi Yulu tea samples. (**B**) Sensory traits and score.

Figure 1A presents the appearance and color of the three Enshi Yulu tea samples derived from the tea cultivars Longjing 43 (EY-L), Xiapu Chunbolv (EY-C), and Zhangcha 108 (EY-Z). Following a sensory evaluation and expert panel discussion, six aroma attributes and five taste attributes were identified, as depicted in Figure 1B. In the evaluation of aroma, “clean and refreshing” along with “pure” emerged as prominent descriptors, with EY-C receiving the highest score. In comparison to other steamed green teas, EY-L exhibited a distinctive chestnut flavor, while EY-Z evoked a stronger fresh odor note. Additionally, the evaluation of high and flowery aromas at lower intensity revealed no significant differences among the three tea samples.

For taste evaluation, the prevailing taste profiles observed across all sample variations were “kokumi” and “mellow”. The intensity of these characteristics varied among the tea varieties, with the “kokumi” trait particularly demonstrating a discernible gradient from robust to delicate, following the sequence of EY-L, EY-Z, and EY-C. It was also noteworthy that EY-Z exhibited more astringency compared to the other two teas, likely attributable to its varietal characteristics. Furthermore, the attributes of umami and strong taste were consistently identified in all three tea samples, albeit at low intensity.

### 2.2. Overall Chemical Profiles of Enshi Yulu

**Figure 2:**
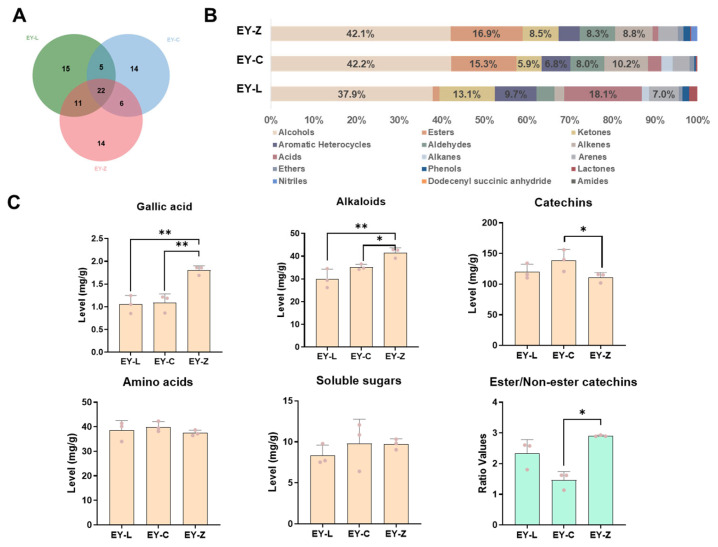
Overall chemical profiles of Enshi Yulu. (**A**) Venn diagram of volatile compounds. (**B**) Relative content percentage of volatile composition. (**C**) Total content/ratio of non-volatile compounds. * and ** represent significant difference (*p* < 0.05) and extremely significant difference (*p* < 0.01), respectively.

The results presented in Figure 2A depict the volatile components of the three tea cultivars, and 87 volatile compounds were detected in all tea samples, with approximately 1/4 (21) of these volatile components being commonly shared among them. The number of varietal-specific components was 16 (EY-L), 16 (EY-C), and 14 (EY-Z), respectively. To provide a comprehensive overview of the volatile components, a stacking diagram was created, categorizing the aroma components (Figure 1B). Notably, alcohols constituted the majority in Enshi Yulu green tea’s aromatic profile, accounting for over 2/5 of the total composition. EY-Z and EY-C showed a higher percentage of esters (16.9% and 15.3%, respectively), while EY-L had a lower percentage of esters (<5%) but exhibited higher percentages of ketones (13.1%) and acids (18.1%).

The content of non-volatile compounds in tea, including gallic acid, alkaloids, catechins, amino acids, soluble sugars, and the ratio of ester/non-ester catechins is illustrated in Figure 2C. Amino acids, alkaloids, and catechins are widely acknowledged as crucial contributors to the flavor profile of tea infusion [15]. However, among the three tea cultivars, there are no significant differences in amino acids and soluble sugars. In terms of gallic acid content, EY-Z surpassed EY-L and EY-C significantly, while no marked variance was observed between EY-L and EY-C. Additionally, EY-Z showcased notably elevated levels of alkaloids compared to both EY-C and EY-L, which are known to be an important direct source of astringency and bitterness in the tea infusion [16]. Regarding catechin content, EY-C demonstrated a significantly higher level than EY-Z, though no substantial discrepancy was noted between EY-C and EY-L. Of particular significance is the ratio of ester catechins to non-ester catechins, a paramount parameter influencing the briskness and umami, which was significantly higher in EY-Z compared to EY-C.

### 2.3. PLS-DA Analysis of Volatile Compounds for Discriminating Three Cultivars

**Figure 3:**
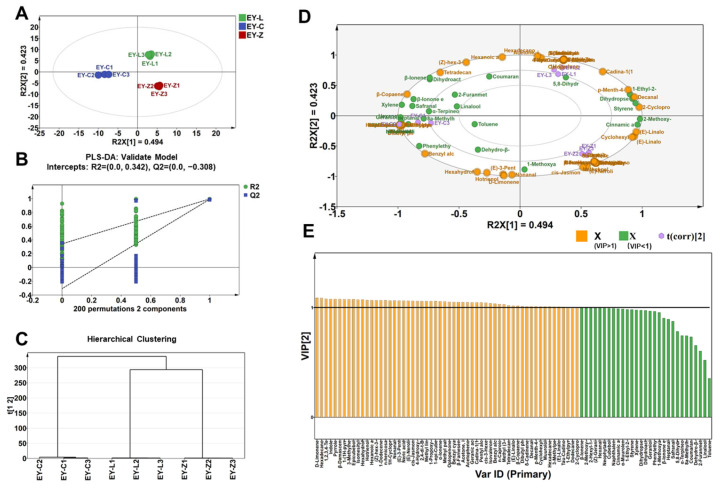
Characteristic volatile components. (**A**) PLS-DA score plot (R2 = 0.917; R2Y = 0.993; Q2 = 0.982). (**B**) Permutation test plot of PLS-DA. (**C**) HCA plot. (**D**) PLS-DA biplot. (**E**) VIP plot of volatile chemical constituents.

EY-L, EY-C, and EY-Z were clearly distinguished using PLS-DA, revealing variations in volatile composition among the different tea cultivars. The extracted first two principal components can explain more than 90% of the variation. EY-L, EY-C, and EY-Z were situated in quadrants 1, 3, and 4, respectively (Figure 3A), which exhibited significant differentiation. Through a permutation test involving 200 calculations, cross-validation outcomes were obtained (Figure 3B). The model demonstrated the intercepts of R2 and Q2, which were 0.342 and −0.308, respectively, confirming its reliability and ruling out overfitting. Furthermore, the results of HCA suggested that EY-L and EY-Z exhibited a higher degree of similarity (Figure 3C), which aligned with the fact that Zhongcha 108 was derived from Longjing 43 through irradiation [17]. The biplot (Figure 3D) provides a detailed representation of the clusters associated with volatile compounds related to EY-C, EY-L, and EY-Z. To further clarify the specific differences in aroma composition among cultivars, key variables were screened based on the criterion of variable importance in the projection (VIP) >1 and *p* < 0.05 (Figure 3E). This screening process aimed to identify the significant variables contributing to the discrimination among the cultivars, allowing for a more detailed understanding of their aroma composition.

### 2.4. Characteristic Volatile Composition Analysis

**Figure 4:**
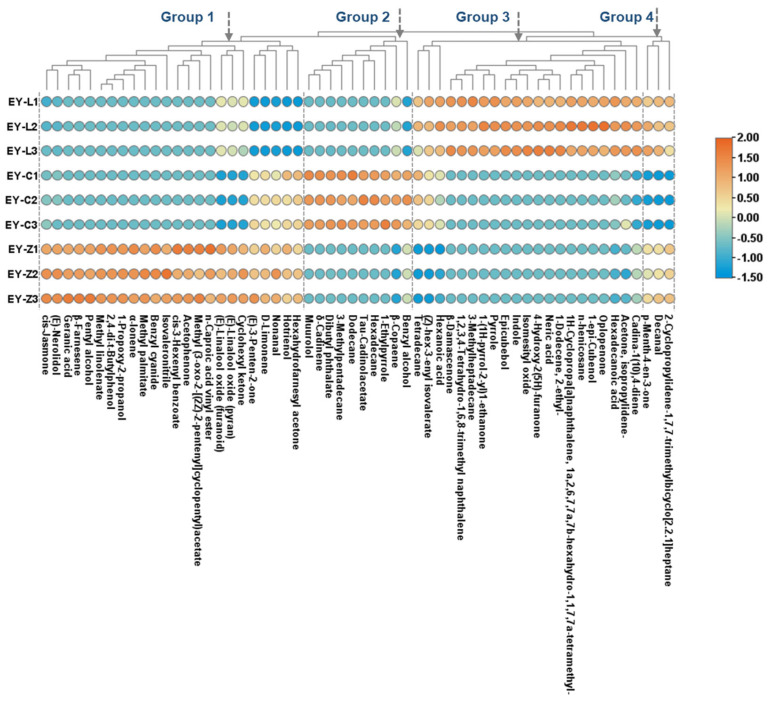
Clustering heat map of characteristic volatile components.

A total of 58 distinct volatile compounds were identified, including 11 ketones, 10 alcohols, 9 esters, 8 alkenes, 6 alkanes, 3 acids, 3 aromatic heterocycles, 2 arenes, 2 aldehydes, 2 nitriles, 1 lactone and 1 phenol. These compounds were subjected to heat map clustering analysis, resulting in the formation of four noticeable aroma groups (Figure 4). Group 1 consisted of 24 volatile compounds closely associated with EY-Z, predominantly evoking floral or green notes, such as acetophenone, (E)-nerolidol, cis-jasmone, (E)-linalool oxide (furanoid), hexahydrofarnesyl acetone, cis-3-hexenyl benzoate, and geranic acid. Additionally, hexahydrofarnesyl acetone, hotrienol, nonanal, D-limonene, and (E)-3-penten-2-one were also identified in EY-C, albeit at lower levels. Cyclohexyl ketone, (E)-linalool oxide (pyran), and (E)-linalool oxide (furanoid) were found in EY-L, but in smaller quantities. Group 2 comprised 10 components specifically linked to EY-C, primarily sesquiterpenes and their derived alcohols and esters, such as β-copaene, δ-cadinene muurolol, and τ-cadinol acetate. Group 3 encompassed compounds predominantly associated with EY-L, including 21 substances. Some acids involved can evoke a waxy odor, such as hexadecanoic acid, and some ketones, like acetone, isopropylidene, and isomesityl oxide, elicited vegetable odor notes. Group 4 contained only three compounds identified in both EY-L and EY-Z, indicating the similarity of the two tea cultivars.

### 2.5. Differentiated Non-Volatile Components Analysis

**Figure 5:**
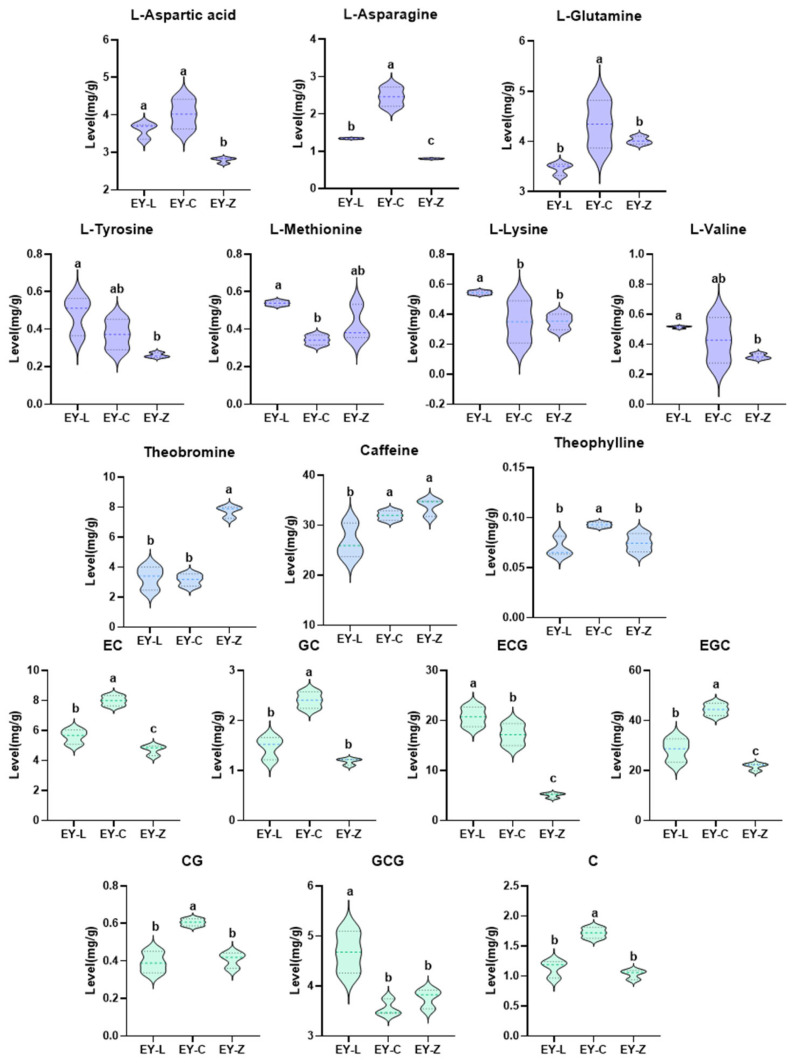
Non-volatile compounds with significant differences in content. Different letters indicate statistically significant differences among samples (*p* < 0.05).

Further analysis screened 18 differentiated non-volatile components through one-way ANOVA, including 7 amino acids, 7 catechins, 3 alkaloids, and gallic acid (Figure 5). For all amino acids, both EY-L and EY-C exhibited heightened concentrations compared to EY-Z. Notably, EY-C stood out with significantly elevated levels of L-aspartic acid, L-asparagine, and L-glutamine, while EY-L showcased notably higher content of L-tyrosine, L-methionine, and L-lysine. Among the alkaloids, EY-Z featured significantly higher levels of theobromine and caffeine compared to the other two cultivars. Regarding catechins, EY-C presented significantly greater concentrations of non-ester catechins than the other two varieties. EY-L demonstrated a marked prominence in ECG and GCG, whereas EY-Z exhibited relatively lower overall catechin content. The results obtained from the SPSS analysis align harmoniously with previous sensory evaluations and analyses, thereby further refining our understanding of these taste distinctions.

### 2.6. Association between Characteristic Chemical Contributors and Sensory Traits

**Figure 6:**
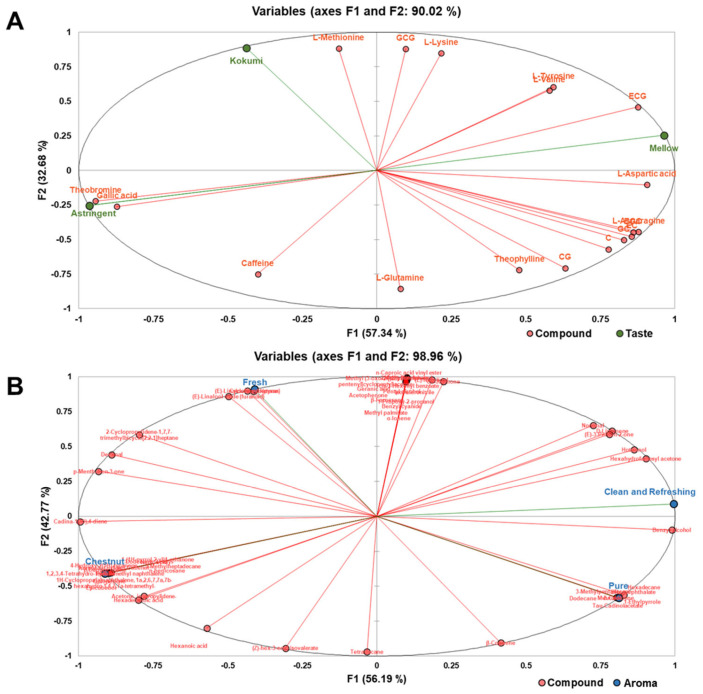
MFA-loading plot of sensory traits and key chemical contributors. (**A**) Taste traits and characteristic non-volatile compounds. (**B**) Aroma traits and characteristic volatile compounds.

The MFA plot provides a vivid illustration of the interrelation between non-volatile components and the three distinct taste attributes—kokumi, mellow, and astringency (Figure 6A). The principal component factors F1 accounted for 57.34%, and F2 accounted for 32.68%, contributing 90.02% of the cumulative variance. Theobromine and gallic acid showcase a positive correlation with “astringent” within the third quadrant while revealing a negative correlation with “mellow” within the first quadrant. The result elucidated the source of astringency characterizing the EY-Z sample, effectively distinguishing it from the other two varieties. The score plot also reveals that several amino acids, including L-aspartic acid, L-asparagine, L-tyrosine, and L-valine, and catechins such as EGC, EC, and ECG contributed to the “mellow” taste attribute, implying that an increase in these substances might be beneficial in enhancing the mellowness of the tea infusion. In addition, L-methionine and GCG exhibit a strong correlation with “kokumi”, which was notably high in EY-L.

Figure 6B also visualizes the potential link between the key variable volatile compounds and the aroma quality. Within the first two principal components extracted, F1 accounted for 56.19% of the variance of the dataset, and F2 accounted for 42.77%, collectively contributing to a cumulative variance of 98.96%. Among the EY-L-associated volatiles (Group 3 and Group 4 in Figure 5), the majority (20/24) were positively correlated with “chestnut” aroma. Volatiles such as δ-cadinene, hexadecanoic acid, β-copaene, and D-limonene were prominently positioned in the “pure” direction, suggesting their potential role as pivotal constituents contributing to a “pure” aroma profile. Additionally, there are 14 volatile components showing a positive correlation with the “clean and refreshing” aroma, with D-limonene, hexahydrofarnesyl acetone, hotrienol, (E)-3-penten-2-one, and benzyl alcohol having coefficients above 0.8. Volatile compounds such as (E)-nerolidol, cis-jasmone, 2,4-di-t-butylphenol, methyl linolenate, 1-propoxy-2-propanol, methyl palmitate, benzyl cyanide, β-farnesene, acetophenone, geranic acid, pentyl alcohol, cis-3-hexenyl benzoate, methyl (3-oxo-2-[(2Z)-2-pentenyl] cyclopentyl) acetate, (E)-linalool oxide (pyran), decanal, cyclohexyl ketone, and (E)-linalool oxide (furanoid) were positively correlated with “fresh” odor. The relative content and detailed information of these compounds are presented in Table 1 and Table 2.

## 3. Discussion

### 3.1. Organoleptic Quality and Chemical Profiles of Enshi Yulu

For taste attributes, Enshi Yulu retained the similar umami, mellow, astringency, and kokumi characteristics as most green teas [16], which was attributed to the content of non-volatile components like catechins, amino acids, and soluble sugars. Previous studies have suggested that the expression of these two characteristics, “kokumi” and “mellow”, could be related to higher levels of tea polyphenol substance content [18]. Although the taste attributes are shared in most green teas, the intensity may differ due to the disparity in the content of these metabolites [19]. Additionally, aroma stands out as a paramount criterion in evaluating tea quality [20], and the aroma formation of green tea is closely related to the manufacturing technology applied, especially the fixation and drying [7]. In this study, steaming fixation was adopted and facilitated the fresh, clean, and refreshing aroma of Enshi Yulu, which has been proven to be easily formed during steam fixation with penetrability [21]. Compared to other green teas produced by microwave, pan-frying, or hot air, steamed green teas exhibited a unique, pure, and clean aroma [22,23]. These also aligned with the distinctive attributes of high-quality green teas, known for their essence of freshness, purity, and aromatic delicacy [24]. Similar to the pan-fried green tea [25] and Japanese steamed green tea [9], alcohols constituted the predominant share in Enshi Yulu green tea’s aromatic profile, which was much higher than the percentage of baked green tea (about 25%) [4]. However, the total abundance of volatiles in steamed green teas was typically lower [4], along with the distinctive volatile composition contributing to the invigorating aromatic character distinctive to Enshi Yulu tea. 

### 3.2. Characteristic of the Three Tea Cultivars Processing Enshi Yulu

The varietal characteristics of Enshi Yulu were reflected in the taste sensory intensity and non-volatile profiles. Amino acids mainly provided umami flavor and could effectively mitigate the bitterness and astringency of green tea infusion [2,18], while soluble sugars provided sweetness [26] and also acted as precursors to some aroma substances [27]. Although no significant differences were observed in total amino acids and soluble sugars among the three tea cultivars, their composition varied distinctly. L-aspartic acid and L-glutamate are acidic amino acids known to enhance the umami taste [28], but the higher content of the two compounds in EY-L and EY-C resulted in no disparity in sensory evaluation of umami taste, probably due to the lower content of gallic acid compared to EY-Z, which has been recognized as umami-enhancing compound [16]. Theobromine, caffeine, and theophylline are the major alkaloids in tea, with caffeine and theobromine found at higher levels. Among the three cultivars, EY-L exhibited the lowest levels of caffeine and theobromine. Previous studies have suggested that ester catechins constitute the primary source of bitterness in tea infusion [8,18]. Remarkably, the ratio of ester/non-ester catechins was significantly higher in EY-Z compared to EY-C, aligning with the astringency evaluation determined through sensory analysis. Regarding catechin content and composition, EY-C was distinguished from EY-L and EY-Z by the high content of non-ester catechins. This disparity could be attributed to the early budding prosperity, which likely favored the accumulation of gene expression levels involved in catechin metabolism [29].

The three tea cultivars shared 22 volatile compounds, primarily including 6 alcohols, 6 ketones, 2 esters, 2 arenes, 2 aldehydes, 1 aromatic heterocycle, 1 lactone, 1 phenol, and 1 ether. Notably, coumaran, linalool, and 2-furanmethanol exhibited relatively high relative content across all tea cultivars. Coumaran has been proven to contribute to a typical green tea odor [30]. The 2-furanmethanol can evoke burnt and caramel odors in black teas, but its high odor threshold may limit its perceptibility [31]. Linalool found widely in green teas, can be derived from both the primary oxidation of carotenoids and the hydrolysis of glycosides [27]. The sensory evaluations indicated that the three samples had common qualities of Enshi Yulu, but they also exhibited differentiated characteristics, which could be attributed to varietal differences.

EY-Z displayed distinct characteristics with 14 unique volatile components, primarily comprising esters. The most notable constituent in EY-Z was methyl linolenate, which exhibited a relatively subdued aromatic intensity. Additionally, two nitriles, an L-leucine-derived nitrogen-containing compound isovaleronitrile [32] and aromatic benzyl cyanide, were also identified in EY-Z. EY-C encompassed 14 unique volatiles, mainly terpenes and their derived alcohols, such as δ-cadinene, (Z)-geraniol, muurolol, and cubebol. Notably, alkanes, including dodecane, 3-methylpentadecane, and hexadecane, accounted for a significant proportion of the volatile profile in EY-C.

EY-L exhibited a distinct volatile composition comprising 15 unique components, with ketones and aromatic heterocycles being the prominent chemical types, such as β-damascenone, indole, pyrrole, 1-(1H-pyrrol-2-yl)1-ethanone. Typically, β-damascenone, derived from carotenoid degradation, possessed an exceptionally low aroma threshold (0.002 ppm). Indole and pyrrole are known to arise from the Maillard reaction [33], and another possible precursor of indole is tryptophan, which can be oxidized by tryptophan indole-lyase [34].

### 3.3. The Relationship between Sensory Attributes and Chemical Contributors

In the correlation analysis of non-volatile compounds and taste characteristics of Enshi Yulu, specific taste attributes were found to have positive correlations with various amino acids, catechins, and alkaloids. Notably, amino acids L-aspartic acid, L-asparagine, L-tyrosine, and L-valine, along with catechins EGC, EC, and ECG, showed positive correlations with the “mellow” taste. These amino acids, which are constituents of proteins, are known to contribute to the umami and mellow taste [35]. Hence, the level of free amino acids is considered an important indicator of tea quality. The content of non-ester catechins EGC and EC were higher in EY-C, with EGC exhibiting significantly higher concentrations compared to other non-ester catechins. Furthermore, ECG, the catechin identified as emitting the highest taste intensity [36], potentially has a more significant influence on tea taste perception than anticipated. Astringency, a crucial sensory property of green tea, was positively correlated with gallic acid and theobromine, both of which were higher in EY-Z. Gallic acid can accumulate through the degradation of ester catechins, and certain phenolic acids, including gallic acid, contribute to the astringent taste [2]. Theobromine, a prominent purine alkaloid in teas, can enhance the bitterness and astringency of the tea infusion [16]. The “kokumi” flavor was associated with L-methionine, L-lysine, and GCG. Kokumi is a taste sensation distinct from the five primary taste attributes mediated by its receptor called the calcium-sensing receptor (CaSR), and some peptides have been identified as key contributors to this flavor sensation [37]. GCG, which is not typically present in fresh tea leaves but is produced during tea processing, likely contributes to the “kokumi” flavor in this study.

Multiple volatile compounds were identified as positively correlated with the “clean and refreshing” (14), “pure” (10), “chestnut” (19), and “fresh” (21) aromas, respectively. The correlations aligned with the distribution of the aroma group, as shown in Figure 4.

Regarding the volatile components associated with “clean and refreshing”, the prominent constituents encompassed D-limonene, hexahydrofarnesyl acetone, nonanal, hotrienol, (E)-3-penten-2-one, benzyl alcohol, dibutyl phthalate, δ-cadinene, muurolol, τ-cadinol acetate, and 1-ethylpyrrole. Among them, D-limonene has a lemon-like aroma as one of the main aroma components in citrus plants [38] and has been proven to evoke fresh, citrusy [39], and grassy [5] aromas in green teas. Similarly, nonanal, which possesses grassy, citrus, and green characteristics, emerged as a noteworthy aroma-active component in green tea [40]. It primarily originated from the degradation of unsaturated fatty acids like oleic acid and palmitoleic acid [40,41,42]. Furthermore, (E)-3-penten-2-one was reported to emit an ethereal-fruity odor [43], and hotrienol liberated from linalool glycosidic precursors evoked a flowery aroma. Additionally, benzyl alcohol, dibutyl phthalate, δ-cadinene, muurolol, τ-cadinol acetate, and 1-ethylpyrrole were also identified as “fresh” odor contributors. Benzyl alcohol presented a notably high relative content in both EY-C and EY-Z and is characterized by its sweet, floral (rose-like) aroma [44]. This aromatic alcohol is commonly found in a variety of teas and can be liberated through glycoside hydrolysis [44,45,46]. Dibutyl phthalate emerged as the most abundant compound, also recognized as a key aroma constituent in Meitan Cuiya [47]. Sesquiterpene δ-Cadinene is an important contributor to the aroma of chrysanthemum flowers [48] and has been detected in a variety of essential oils [49,50]. δ-Cadinene and muurolol also acted as active ingredients in the volatile substances of some herbal essential oils [51]. The 1-Ethylpyrrole with a burnt odor note can be attributed to the reaction between theanine and D-glucose or other monosaccharides above 150 °C [27]. In spite of the above-mentioned volatile compounds, β-copaene, considered one of the aroma contributors to the Chinese herb Panax notoginseng flowers [52], was also identified as responsible for the “pure” flavor. 

In the case of volatile components associated with the “Chestnut” aroma, two major aromatic heterocycles, indole (animal-like) and pyrrole (nutty), were identified in EY-L. Indole has the property of changing the odor from an unpleasant odor to a floral scent aroma as the dilution factor increases [33] and has previously been reported to enhance the aroma in Japanese green tea [53]. Similarly, pyrrole has been linked to the roasted and chestnut aromas of teas in other scientific investigations [54,55]. Furthermore, terpenes and terpene derivatives cadina-1(10),4-diene, β-oploplenone, 1-epi-cubenol, and epi-cubebol were also involved, inducing woody, herbal odor notes [56]. Additionally, the related volatile compounds also contained some acids like neric acid and hexadecanoic acid, as well as ketones such as β-damascenone, isomesityl oxide, isobutenyl methyl ketone, and p-menth-4-en-3-one. β-Damascenone, in particular, has been proven to provide sweet-like aromas such as floral [57], fruity [58], cooked apple [59], and woody [60], making it an important aroma contributor in Dianhong and green tea [20,60].

For the “fresh” aroma contributors, linalool oxides played a significant role in the aroma profile of tea. These compounds were primarily formed through glycoside hydrolysis in enzymatic reactions [27]. (E)-linalool oxide (pyran) has been described as having a tree-root and grassy aroma, in addition to its floral characteristics [33,61]. On the other hand, (E)-linalool oxide (furanoid), despite its lower concentration, provided a floral, woody odor [62]. Two distinct compounds, (E)-nerolidol and cis-jasmone, were two typical contributors to floral aromas, with the former derived from carotenoid, providing mainly neroli-like, rose-like, and sweet odors [63], and the latter produced through lipid degradation, predominantly provided notes of jasmine and herbal scents [44,64]. Additionally, decanal, a key contributor to grassy and green odors [41], can interact with other primary aroma contributors to modulate scent [40].

## 4. Materials and Methods

### 4.1. Tea Sample Preparation

Three Enshi Yulu samples were prepared using the fresh leaves of tea cultivar Longjing 43 (EY-L), Chunbo Lv (EY-C), and Zhongcha 108 (EY-Z) in Enshi, Hubei. According to the technological regulations for Enshi Yulu processing (DB42/T611), fresh tea leaves of one bud with one leaf were picked by hand in April 2022. After a natural withering period of 8 h, the leaves underwent a 50 s steaming process at a steam pressure of 3.8–4.0 Mpa and a hot air temperature of 520–550 °C. Subsequently, the steamed leaves were fixed at 120 °C for 15 min, followed by rolling for 40 min using the “light–heavy–light” pressure standard. The rolled tea leaves were then subjected to drying at 110 °C, 100 °C, and 90 °C for 45 min, 30min, and 45min, respectively, with intervals to ensure moisture uniformity. Shaping was also completed during the above drying process, and the final moisture content of three tea samples was approximately 5%. Tea samples for further investigations were obtained through the uniform sampling of each kind of processed tea. 

### 4.2. Reference Chemical Standards

The reference chemical standards for free amino acids (≥98%) were sourced from Yuanye Bio-Technology Co., Ltd. (Shanghai, China). These standards contained L-glutamic acid, L-aspartic acid, L-asparagine, L-serine, L-glutamine, L-histidine, L-methionine, L-tryptophan, L-glycine, L-phenylalanine, L-arginine, L-theanine, L-alanine, L-aminobutyric acid, L-tyrosine, L-threonine, L-valine, L-isoleucine, L-leucine, and L-lysine. Gallic acid, alkaloids including caffeine, theobromine, and theophylline, and catechins including (−)-gallocatechin (GC), (−)-epigallocatechin (EGC), (+)-catechin (C), (−)-epicatechin (EC), (−)-epigallocatechin gallate (EGCG), (−)-gallocatechin gallate (GCG), (−)-epicatechin gallate (ECG), and (−)-catechin gallate (CG) were purchased from Aladdin (Shanghai, China).

### 4.3. Non-Volatile Components Determination

A precise amount of tea powder (0.15 g) with a diameter of less than 0.45 mm was extracted using 25 mL of 50% ethanol. The solutions were heated at 70 °C for 30 min, followed by centrifugation at 12,000 r/min for 15 min. The supernatant obtained after centrifugation was used for the detection and analysis of non-volatile compounds. Each tea sample was repeatedly extracted and evaluated three times.

The amino acid content was determined utilizing HPLC (LC-20A, Shimadzu, Tokyo, Japan) with a fluorescence detector and an Agilent Eclipse-AAA Column (4.6 mm × 150 mm, 3.5 µm) maintained at 40 °C. The pre-column derivatization method with ortho-phthalaldehyde (OPA) was employed. Briefly, a uniform mixture to be investigated was prepared by combining 5 µL of previously prepared sample extracts, 500 µL of 0.4 mol/L boric acid buffer (pH 10.2), 50 µL of a 10 mg/mL OPA solution, and 450 µL of deionized water. The HPLC mobile phases consisted of a 40 mmol/L Na_2_HPO_4_ solution (A) and a mixture of 45% acetonitrile, 45% methanol, and water (B). Reference chemical standards for free amino acids were used in the detection for absolute determination. The injection volume was set at 10 µL, and the flow rate was maintained at 1.5 mL/min. The gradient elution method employed the following conditions: 20% phase B from 0 to 1.9 min; 20%~57% phase B from 1.9 to 18.1 min; 57%~100% phase B from 18.1 to 18.6 min; 100% phase B from 18.6 to 22.3 min; 100%~5% phase B from 22.3 to 23.2 min; and 5% phase B (23.2~30.0 min). 

The detection of catechins and alkaloids was performed using HPLC-UV with Agilent TC-C18 columns (Santa Clara, CA, USA, 4.6 mm × 250 mm, 5 µm), maintained at 25 °C. The HPLC mobile phases were 3% acetonitrile/0.5% acetic acid/water (A) and 30% acetonitrile/0.5% acetic acid/water (B). Reference chemical standards for catechins and alkaloids were also used in the detection for absolute determination. The injection volume was set at 10 µL, and the flow rate was maintained at 1 mL/min. The gradient elution method was as follows: 20% B ramped linearly to 75% within 40 min, then decreased to 20% within the 40–45 min interval and was kept constant at 20% B for 45–50 min. 

The soluble sugar content was detected by the anthrone colorimetric method [65]. The tea sample was treated with sulfuric acid and anthrone, resulting in the formation of a green complex. The absorption value of the complex was measured at a wavelength of 625 nm. 

### 4.4. Extraction and Identification of Volatile Composition

Aroma compounds were extracted utilizing a modified simultaneous distillation extraction method [66]. A total of 250 mL boiling water and 30 mL ether where an appropriate amount of butylated hydroxytoluene (BHT) has been added as internal standard were applied as a solvent to extract 15 g of tea samples in a Liens–Nickerson simultaneous steam distillation continuous extraction for 120 min. The extraction process was carried out at a boiling point to facilitate the continuous evaporation of water and ether. The ether phase containing volatile compounds was subsequently dehydrated with anhydrous sodium sulfate overnight. Then, the dehydrated ethyl ether was concentrated to 5 mL for GC-MS analysis on a Shimadzu gas chromatograph 2010-plus with a triple quadrupole mass spectrometer QP2020 (Shimadzu, Shanghai, China). Each tea sample was repeatedly extracted and analyzed three times. The SH-Rxi-5Sil MS capillary column (30 m × 0.25 μm × 0.25 μm) was employed, and the temperature of the injection port was set to 250 °C with splitless mode. High-purity helium (>99.999%) was adopted as carrier gas with a flow rate of 1.0 mL/min, and the injection volume was 1.0 µL. 

The oven temperature was initially set at 50 °C and held for 5 min, then increased to 210 °C at the rate of 3 °C/min and held for 5 min, and finally increased to 230 °C at the rate of 15 °C/min and held for 5 min. The mass spectrometer conditions were as follows: the ion source temperature was set to 250 °C, and the electron energy was set to 70 eV. The full scan range covered 35–450 atomic mass units with a solvent delay of 3 min.

### 4.5. Sensory Evaluation

According to the “Methodology of sensory evaluation of tea” of national standards (GB/T 23776–2018), the tea sample (3.0 g) was brewed in water (150 mL) at 100 °C for 4 min. A professional and qualified sensory panel (five men and three women, aged between 24 and 50) was invited to describe and score the attributes of the tea samples using quantitative descriptive analysis. Each sensory attribute was scored on a 5-point scale three times.

### 4.6. Statistical Analysis

All experiments were conducted in triplicate to ensure the reliability of the results. Variance analysis (ANOVA) was performed using IBM SPSS Statistics software (Version 25.0, SPSS Inc., Chicago, IL, USA) to assess the statistical significance of the data. Partial least squares discriminant analysis (PLS-DA) and hierarchical cluster analysis (HCA) were executed using SIMCA 14.1 software (Umetrics AB, Umea, Sweden) for further analysis and modeling. To examine the relationship between sensory attributes and chemical compounds in Enshi Yulu samples, multiple factor analysis (MFA) was employed using XLSTAT 2019 (Addinsoft, New York, NY, USA). GraphPad Prism 8.0.1 (GraphPad Software Corporation, La Jolla, CA, USA) and Origin 2023b (OriginLab Corporation, Northampton, MA, USA) were utilized for data visualization and graph plotting.

## 5. Conclusions

This study has provided a thorough exploration of the overall chemical profiles linked to the organoleptic quality of Enshi Yulu tea samples. Notably, alcohols emerged as the dominant volatile components in Enshi Yulu teas sourced from the three distinct tea cultivars. While sharing some characteristic flavors with general green teas, such as umami, mellowness, astringency, and kokumi attributes, Enshi Yulu stands out with its unique steaming fixation process, endowing it with fresh, clean, and refreshing aroma attributes. The disparities in chemical components among tea cultivars Longjing 43, Xiapu Chunbolv, and Zhongcha 108 were also unveiled. The composition of amino acids, the proportion of ester catechins, and the content difference in alkaloids can be used to distinguish the three cultivars, and the characteristic volatile composition was also illustrated by PLS-DA. Notably, Enshi Yulu tea produced from Xiapu Chunbolv, characterized by its earliest budding property among the three cultivars, exhibited the highest level of non-ester catechins. The chemical factors contributing to the characteristic taste and aroma in the three Enshi Yulu teas were explored by MFA. It was found that the “pure” and “clean and refreshing” flavors shared similar volatile compositions. The findings of this study have significantly enhanced our comprehensive knowledge of the metabolic variation induced by steaming fixation in Enshi Yulu tea production. 

## Figures and Tables

**Table 1:** Volatile compounds associated with aroma attributes screened by MFA.

Compound			Odor	Categories	EY-L1	EY-L2	EY-L3	EY-C1	EY-C2	EY-C3	EY-Z1	EY-Z2	EY-Z3	CAS
					Relative Content									
**Clean and refreshing**		D-Limonene	Citrus	Alkenes	/	/	/	0.162	0.163	0.154	0.219	0.204	0.211	5989-27-5
		Hexahydrofarnesyl acetone	Floral	Ketones	/	/	/	0.025	0.022	0.021	0.020	0.023	0.024	502-69-2
		Hotrienol	Tropical	Alcohols	/	/	/	0.037	0.028	0.030	0.038	0.035	0.031	29957-43-5
		Nonanal	Aldehydic	Aldehydes	/	/	/	0.091	0.111	0.096	0.116	0.171	0.147	124-19-6
		(E)-3-Penten-2-one	/	Ketones	/	/	/	0.011	0.012	0.013	0.013	0.015	0.019	3102-33-8
	**Pure**	Benzyl alcohol	Floral	Alcohols	/	/	/	0.201	0.223	0.183	0.104	0.101	0.099	100-51-6
		Dibutyl phthalate	Faint	Esters	0.007	0.006	0.005	0.383	0.375	0.380	0.016	0.020	0.018	84-74-2
		δ-Cadinene	/	Alkenes	/	/	/	0.043	0.042	0.039	/	/	/	483-76-1
		Muurolol	Herbal	Alcohols	/	/	/	0.015	0.014	0.013	/	/	/	19435-97-3
		Hexadecane	/	Alkanes	/	/	/	0.013	0.014	0.012	/	/	/	544-76-3
		3-Methylpentadecane	/	Alkanes	/	/	/	0.011	0.009	0.011	/	/	/	2882-96-4
		Tau-Cadinolacetate	/	Esters	/	/	/	0.013	0.015	0.012	/	/	/	149197-48-8
		1-Ethylpyrrole	Burnt	Aromatic heterocycles	/	/	/	0.019	0.019	0.023	/	/	/	617-92-5
		Dodecane	/	Alkanes	/	/	/	0.029	0.022	0.025	/	/	/	112-40-3
		β-Copaene	/	Alkenes	0.009	0.007	0.008	0.015	0.017	0.017	/	/	/	18252-44-3
**Chestnut**		Hexadecanoic acid	Waxy	Acids	0.377	0.373	0.373	0.082	0.105	0.070	/	/	/	57-10-3
		1,2,3,4-Tetrahydro-1,6,8-trimethyl naphthalene	/	Arenes	0.005	0.005	0.005	/	/	/	/	/	/	30316-36-0
		Indole	Animal	Aromatic heterocycles	0.019	0.021	0.021	/	/	/	/	/	/	120-72-9
		Pyrrole	Nutty	Aromatic heterocycles	0.011	0.011	0.010	/	/	/	/	/	/	109-97-7
		β-Damascenone	Floral	Ketones	0.005	0.005	0.006	/	/	/	/	/	/	23726-93-4
		1-(1H-pyrrol-2-yl)1-ethanone	Musty	Ketones	0.120	0.138	0.116	/	/	/	/	/	/	1072-83-9
		3-Methylheptadecane	/	Alkanes	0.006	0.005	0.005	/	/	/	/	/	/	6418-44-6
		Epicubebol	/	Alcohols	0.008	0.009	0.009	/	/	/	/	/	/	38230-60-3
		Isomesityl oxide	Vegetable	Ketones	0.004	0.005	0.005	/	/	/	/	/	/	673542
		(4E)-4-Hexenyl hexanoate	/	Esters	0.023	0.025	0.021	0.016	0.015	0.016	0.007	0.009	0.010	/
		1-Dodecene, 2-ethyl-	/	Alkenes	0.004	0.005	0.005	/	/	/	/	/	/	19780-34-8
		1H-Cyclopropa[a]naphthalene, 1a,2,6,7,7a,7b-hexahydro-1,1,7,7a-tetramethyl-	/	Alkenes	0.009	0.012	0.008	/	/	/	/	/	/	154098-14-3
		Neric acid	Green	Acids	0.014	0.018	0.020	/	/	/	/	/	/	4613-38-1
		4-Hydroxy-2(5H)-furanone	/	Lactones	0.027	0.036	0.040	/	/	/	/	/	/	541-57-1
		1-epi-Cubenol	/	Alcohols	0.009	0.013	0.009	/	/	/	/	/	/	19912-67-5
		Oplopenone	/	Ketones	0.007	0.009	0.006	/	/	/	/	/	/	28305-60-4
		Isobutenyl methyl ketone	Vegetable	Ketones	0.014	0.015	0.014	0.010	0.010	0.012	0.010	0.009	0.009	141-79-7
		Cadina-1(10),4-diene	/	Alkenes	0.021	0.024	0.025	/	/	/	0.010	0.009	0.008	16729-01-4
		p-Menth-4-en-3-one	/	Ketones	0.014	0.018	0.019	/	/	/	0.012	0.010	0.013	5113-66-6
**Fresh**		(E)-Nerolidol	Floral	Alcohols	0.007	0.007	0.008	0.015	0.016	0.010	0.068	0.077	0.077	40716-66-3
		cis-Jasmone	Floral	Ketones	0.027	0.029	0.031	0.032	0.034	0.035	0.058	0.062	0.063	488-10-8
		2,4-di-t-Butylphenol	/	Phenols	/	/	/	/	/	/	0.006	0.006	0.006	96-76-4
		Methyl linolenate	Oily	Esters	/	/	/	/	/	/	0.387	0.383	0.383	301-00-8
		1-Propoxy-2-propanol	Mild Ether	Alcohols	/	/	/	/	/	/	0.039	0.041	0.041	1569-01-3
		Ionene	/	Arenes	/	/	/	/	/	/	0.005	0.005	0.004	475-03-6
		Methyl palmitate	Waxy	Esters	/	/	/	/	/	/	0.014	0.017	0.016	112-39-0
		Benzyl cyanide	Aromatic	Nitriles	/	/	/	/	/	/	0.022	0.022	0.017	140-29-4
		β-Farnesene	Woody	Alkenes	/	/	/	/	/	/	0.006	0.006	0.008	18794-84-8
		Acetophenone	Floral	Ketones	/	/	/	/	/	/	0.022	0.016	0.019	98-86-2
		Geranic acid	Green	Acids	/	/	/	/	/	/	0.019	0.021	0.025	459-80-3
		Pentyl alcohol	Fermented	Alcohols	/	/	/	/	/	/	0.035	0.034	0.045	71-41-0
		cis-3-Hexenyl benzoate	Green	Esters	/	/	/	/	/	/	0.008	0.006	0.007	25152-85-6
		Isovaleronitrile	/	Nitriles	/	/	/	/	/	/	0.017	0.025	0.019	625-28-5
		n-Caproic acid vinyl ester	/	Esters	/	/	/	/	/	/	0.008	0.006	0.005	3050-69-9
		Methyl (3-oxo-2-[(2Z)-2-pentenyl]cyclopentyl)acetate	Floral	Esters	/	/	/	/	/	/	0.011	0.007	0.010	42536-97-0
		(E)-Linalool oxide (pyran)	/	Alcohols	0.062	0.057	0.060	/	/	/	0.112	0.109	0.120	39028-58-5
		Decanal	Aldehydic	Aldehydes	0.006	0.005	0.006	/	/	/	0.004	0.004	0.005	112-31-2
		Cyclohexyl ketone	Minty	Ketones	0.010	0.010	0.008	/	/	/	0.016	0.020	0.019	119-60-8
		(E)-Linalool oxide (furanoid)	Floral	Alcohols	0.017	0.016	0.016	/	/	/	0.030	0.024	0.029	34995-77-2
		2-Cyclopropylidene-1,7,7-trimethylbicyclo[2.2.1]heptane	/	Alkenes	0.008	0.007	0.006	/	/	/	0.007	0.006	0.007	/

Data of “odor” from http://www.perflavory.com/ or https://pubchem.ncbi.nlm.nih.gov/ (accessed on 12 May 2023).

**Table 2:** Non-volatile compounds associated with taste traits screened by MFA.

Compound		Categories	EY-L1	EY-L2	EY-L3	EY-C1	EY-C2	EY-C3	EY-Z1	EY-Z2	EY-Z3	CAS
			Content (mg/g)									
Mellow	L-Aspartic acid	Amino acids	3.355	3.737	3.700	4.022	3.622	4.423	2.835	2.707	2.833	56-84-8
	L-Asparagine	Amino acids	1.342	1.359	1.325	2.461	2.202	2.719	0.814	0.784	0.800	70-47-3
	L-Tyrosine	Amino acids	0.364	0.512	0.564	0.291	0.454	0.372	0.276	0.250	0.257	60-18-4
	L-Valine	Amino acids	0.517	0.519	0.505	0.276	0.579	0.427	0.338	0.302	0.314	72-18-4
	EGC	Catechins	32.634	23.338	28.698	44.391	46.860	41.922	22.465	22.162	19.816	970-74-1
	EC	Catechins	5.672	5.072	6.045	7.989	8.335	7.644	4.951	4.846	4.312	490-46-0
	ECG	Catechins	20.731	18.752	22.710	14.956	19.367	17.162	5.400	5.204	4.455	1257-08-5
Astringent	Gallic acid	Acids	0.854	1.054	1.247	0.864	1.215	1.188	1.855	1.859	1.690	149-91-7
	Theobromine	Alkaloids	2.454	3.406	4.016	2.720	3.540	3.175	7.887	7.990	7.266	83-67-0
Kokumi	L-Methionine	Amino acids	0.552	0.539	0.526	0.316	0.369	0.342	0.533	0.355	0.381	63-68-3
	L-Lysine	Amino acids	0.544	0.557	0.531	0.209	0.490	0.349	0.400	0.297	0.354	56-87-1
	GCG	Catechins	4.676	4.259	5.094	3.451	3.746	3.467	3.827	3.913	3.543	4233-96-9

## Data Availability

Further information is available from the corresponding authors upon reasonable request.
